# Development and Validation of a Questionnaire to Assess the Radiologists’ Views on the Implementation of Artificial Intelligence in Radiology (ATRAI-14)

**DOI:** 10.3390/healthcare12192011

**Published:** 2024-10-09

**Authors:** Yuriy A. Vasilev, Anton V. Vladzymyrskyy, Yulya A. Alymova, Dina A. Akhmedzyanova, Ivan A. Blokhin, Maria O. Romanenko, Seal R. Seradzhi, Maria M. Suchilova, Yuliya F. Shumskaya, Roman V. Reshetnikov

**Affiliations:** Research and Practical Clinical Center for Diagnostics and Telemedicine Technologies of the Moscow Health Care Department, 127051 Moscow, Russia; npcmr@zdrav.mos.ru (Y.A.V.); vladzimirskijav@zdrav.mos.ru (A.V.V.); alymovaya@zdrav.mos.ru (Y.A.A.); blokhinia@zdrav.mos.ru (I.A.B.); romanenkomo@zdrav.mos.ru (M.O.R.); seradzhisr@zdrav.mos.ru (S.R.S.); suchilovamm@zdrav.mos.ru (M.M.S.); shumskayayf@zdrav.mos.ru (Y.F.S.); reshetnikovrv1@zdrav.mos.ru (R.V.R.)

**Keywords:** artificial intelligence, surveys and questionnaires, radiologists, radiology, attitude toward computers

## Abstract

**Introduction**: Artificial Intelligence (AI) is becoming an essential part of modern radiology. However, available evidence highlights issues in the real-world applicability of AI tools and mixed radiologists’ acceptance. We aimed to develop and validate a questionnaire to evaluate the attitude of radiologists toward radiology AI (ATRAI-14). **Materials and Methods**: We generated items based on the European Society of Radiology questionnaire. Item reduction yielded 23 items, 12 of which contribute to scoring. The items were allocated into four domains (“Familiarity”, “Trust”, “Implementation Perspective”, and “Hopes and Fears”) and a part related to the respondent’s demographics and professional background. As a pre-test method, we conducted cognitive interviews with 20 radiologists. Pilot testing with reliability and validity assessment was carried out on a representative sample of 90 respondents. Construct validity was assessed via confirmatory factor analysis (CFA). **Results**: CFA confirmed the feasibility of four domains structure. ATRAI-14 demonstrated acceptable internal consistency (Cronbach’s Alpha 0.78 95%CI [0.68, 0.83]), good test–retest reliability (ICC = 0.89, 95% CI [0.67, 0.96], *p*-value < 0.05), and acceptable criterion validity (Spearman’s rho 0.73, *p*-value < 0.001). **Conclusions**: The questionnaire is useful for providing detailed AI acceptance measurements for making management decisions when implementing AI in radiology.

## 1. Introduction

Artificial Intelligence (AI) made significant strides in image analysis, progressively improving at processing and interpreting complex data [1,2]. One of the most promising areas of AI application is healthcare, especially radiology [3,4]. The first research on AI in radiology was published in 1983 [5]. In 1998, the U.S. Food and Drug Administration approved the first AI for mammography [6]. However, the widespread implementation of AI in radiology is far from successful. Even though in silico studies generally report high accuracy of medical AI predictions, these studies face well-deserved criticism due to poor design, methodological mistakes, and biased reporting [7]. Furthermore, algorithms trained in “ideal” experimental conditions may experience difficulties transitioning into complex real-world settings [8].

Despite this, policy makers consider AI a promising tool for optimizing healthcare operations, which could help to accelerate radiology reporting, reduce the workload of radiologists, and obtain more accurate and reproducible measurements [9]. Many healthcare organizations are experimenting with integrating AI tools into clinical practice [10]. One of the largest projects of this type in the radiology field started in 2020 in Russia, named “the experiment on the use of innovative computer vision technologies for the medical image analysis and subsequent application in the Moscow healthcare system” (the Moscow Experiment) [11]. The Moscow Experiment is carried out by the Moscow Research and Practical Clinical Center for Diagnostics and Telemedicine Technologies (CDTT) of the Moscow Healthcare Department (MHD) and involves more than 150 medical centers [12]. The major participant of the Moscow Experiment is the clinical department of the CDTT (Moscow Reference Center, MRC [13]), which employs more than 400 radiologists who remotely analyze medical imaging exams for MHD medical centers using AI tools [12].

However, the current results of AI integration into clinical practice are mixed [14]. As the end-users of AI solutions, practicing radiologists might not share the optimistic outlook of policy makers. Unconvinced by its value, some radiologists may develop a negative view of AI and sabotage further adoption. An objective assessment of radiologists’ attitudes toward AI can help identify areas requiring special attention from policy makers and stakeholders.

Several international attempts have been made to develop and apply such assessment tools, reporting overall positive views with a direct relationship between the AI-specific knowledge level and attitude toward AI among radiologists [15,16,17,18,19]. However, the published results might be associated with selection bias since respondents more interested in the topic might be more willing to answer and have a better attitude toward AI [19]. Moreover, these tools lack a scoring system for attitude measurement. Validation and assessment of test–retest reliability, while being the essential steps of a questionnaire’s development [20,21], have not been conducted for any of these tools in any language. Thus, the results of these studies may not fully reflect the true attitude toward AI.

Because of the Moscow Experiment, MHD radiologists have gained extensive experience working with more than 50 AI solutions that address a wide range of medical tasks with varying efficiency [12]. The combined awareness of medical, scientific, and administrative staff involved in the Moscow Experiment provides an appropriate medium for developing a research instrument for collecting objective information about radiologists’ perceptions of AI.

The aim of our study was to develop and validate a questionnaire for the precise measurement of radiologists’ attitudes toward AI and the key factors influencing that attitude.

## 2. Materials and Methods

**Figure 1:**
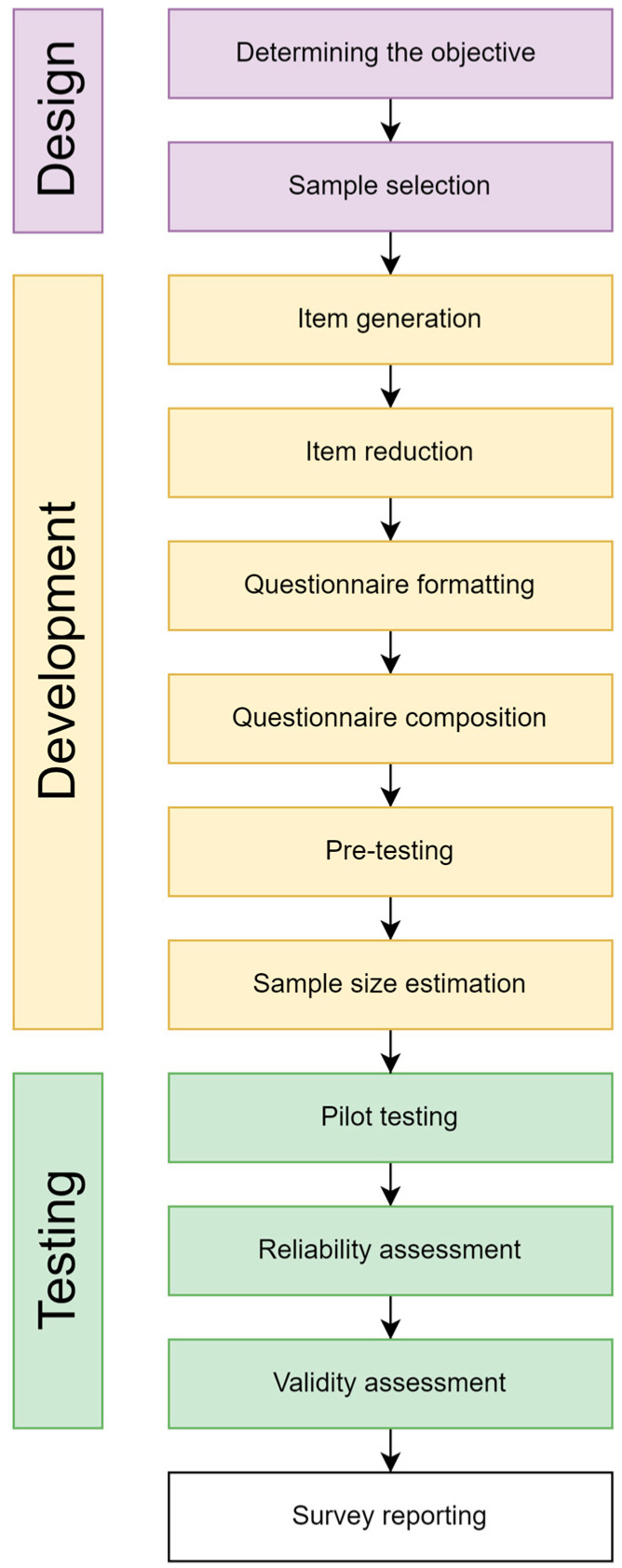
The study flow diagram.

This study was carried out in accordance with the guide for the design and conduct of self-administered surveys of clinicians (Figure 1) [22].

### 2.1. Sample Selection

The target population for the questionnaire is licensed radiologists, radiology residents, and radiology department heads.

### 2.2. Research Settings

The study settings for the questionnaire development were radiology departments of outpatient and inpatient clinics, including a teleradiology center (MRC).

### 2.3. Item Generation

Our questionnaire is based on the European Society of Radiology (ESR) questionnaire, which has 15 items [15]. A multidisciplinary team of survey researchers included four scientists with experience in radiology AI tools and one sociologist. Two professional medical translators performed a linguo-cultural adaptation of the original ESR items to the Russian-speaking population, according to Vasilev et al. [23]. The ESR questions were revised, and the new ones were introduced to match the aim of this study. Items were generated through a combination of in-depth interviews and focus group sessions with experts. The expert group consisted of six radiologists involved in the Moscow Experiment with an average of 8.5 years of work experience. When designing the questionnaire items, we followed the close-ended format to facilitate quantitative analysis through a Likert scale ranging from 1 to 5, corresponding to extremely negative and extremely positive attitudes, respectively.

Some of the questions implied multiple choice. For such questions, the sum of the selected answers’ individual scores was linearly converted to a Likert scale (Appendix A.1, Questions P1, P4, and F6 with corresponding explanations).

Additionally, we have developed a background part to gather demographic and professional information.

### 2.4. Item Reduction

The list of generated items was assessed by an independent focus group of nine radiologists with 5 years of work experience, on average, involved in the Moscow Experiment. Each expert independently chose which question should be included in the final version of the questionnaire. If the majority of experts (five or more) were against the question, it was removed from the final version. Furthermore, focus group members independently assessed the phrasing of each question and made adjustments. The research team decided whether to implement the adjustments after discussion. In the case of five or more similar expert comments, they were accepted without discussion.

### 2.5. Questionnaire Formatting

According to the approach of Cane et al., the behavior of healthcare workers associated with a new technology can be assessed by 14 domains [24]. They include professional characteristics (Professional Role and Identity, Memory and Attention); familiarity with the new technology (Knowledge and Skills, Goals and Intentions, and Reinforcement); trust in the new technology (Beliefs about Capabilities, Optimism); implementation context and perspectives (Environmental Context and Resources, Social Influences, Beliefs about Consequences); and personal factors (Emotions, Behavioral Regulation). We used this approach for our questionnaire domain structure, which consists of the background part followed by the main part consisting of four domains: “Familiarity”, “Trust”, “Implantation Perspectives”, and “Hopes and Fears”.

### 2.6. Questionnaire Composition

For the online questionnaire, we used survey administration software provided by “Yandex.forms”. Questions were presented in a series of linked pages (multiple-item screens) with accompanying electronic instructions.

### 2.7. Pre-Testing

To assess how well respondents understand the items, four survey researchers conducted individual interviews with 20 radiologists similar to the sampling frame. The aim of the interviews was to determine whether the respondents interpreted the question in the way it was intended [25].

### 2.8. Sample Size Estimation

Sample selection for questionnaire validation was performed to be representative of the Moscow radiologists’ population. As of 30 September 2022, there were 1600 radiologists in Moscow, 28.1% of whom were employees of MRC [26]. According to the Sample Size Calculator for Reliability Studies, for the expected Cronbach’s alpha 0.7, precision ±0.1, 95% confidence level, and 16 items, the minimally acceptable sample size should be 79 radiologists (5% of the target population) [27]. To maintain the class balance in the target population, the sample has to include at least 23 MRC radiologists.

### 2.9. Pilot Testing with Reliability and Validity Assessment

Metrics used for reliability and validity assessment are described in Table 1.

Survey researchers with an expert group assessed face validity and content validity. Every member of both teams voted “yes” or “no” on the questions “Does the questionnaire measure what it intends to measure?” and “Does questionnaire content accurately assess all fundamental aspects of the topic?”. If the majority of respondents (eight or more) answered “yes”, the final answer was considered positive.

To assess construct validity, we conducted confirmatory factor analysis (CFA). CFA was performed to test the correspondence between item loadings and the questionnaire domain structure and highlight items requiring revision or removal from a domain.

To assess criterion validity, we performed a correlation analysis of the ATRAI-14 final score with the self-reported visual analogue scale (VAS) score (from 1 to 10, corresponding to extremely negative and extremely positive attitudes, respectively).

Reliability was assessed by test–retest reliability and internal consistency. The sample size for test–retest reliability was calculated according to Vasilev et al., resulting in 20 respondents [23], who filled out the questionnaire twice with a washout period of 14 days in the presence of a survey researcher. Internal consistency was assessed by Cronbach’s alpha evaluation for the main part of ATRAI-14.

### 2.10. Statistical Data Analysis

Data analysis was carried out using the R programming language, v.4.3.1, with additional usage of psych v.2.4.6 [31], lavaan v.0.6-18 [32], ltm v.1.2-0 [33], and ICC v.2.4.0 [34] packages. The Holm–Bonferroni correction was used for multiple comparisons. *p*-value < 0.05 was considered statistically significant for all statistical tests.

## 3. Results

**Figure 2:**
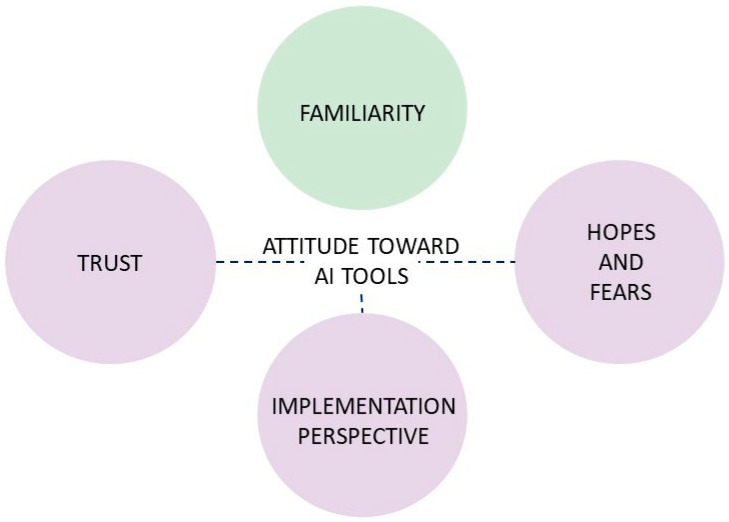
Domain structure of the questionnaire. Dashed lines and colors represent domains contributing to the final score.

We have developed the ATRAI-14 questionnaire to assess radiologists’ attitudes toward the implementation of AI tools. The questionnaire consists of four domains—“Familiarity”, “Trust”, “Implementation Perspective”, and “Hopes and Fears” (Figure 2). The full set of questions may be found in the Appendix A.1 and also on the web page [35].

The “Familiarity” domain aims to evaluate respondents’ personal experience with the AI tools. It considers three areas of experience: clinical practice, development and testing, and participation in clinical trials.

The “Trust” domain evaluates respondents’ perception of the quality and reliability of current AI tools.

The “Implementation Perspective” domain assesses respondents’ perception of policy makers’ and stakeholders’ initiatives regarding AI tool implementation and infrastructure preparedness.

The “Hopes and Fears” domain evaluates respondents’ perception of the potential influence of AI implementation on their personal and career path.

The “Trust”, “Implementation Perspective”, and “Hopes and Fears” domains evaluate different aspects of a respondent’s attitude toward AI tools. The ATRAI-14 final score is a sum of these domains’ weight-adjusted scores (Appendix A.2). The maximum score is 36, and the minimum score is 0; a higher score corresponds to a better attitude. The “Familiarity” domain does not contribute to the total score but provides quantitative data for the survey population pooling by professional experience with AI.

### 3.1. Item Generation and Reduction

We developed four items for the “Familiarity” domain and six items for each domain contributing to scoring (Figure 2). The background part (nine items) gathered demographic and professional information, including AI-using experience. None of the questions required identifying information. The total number of items was 31.

**Figure 3:**
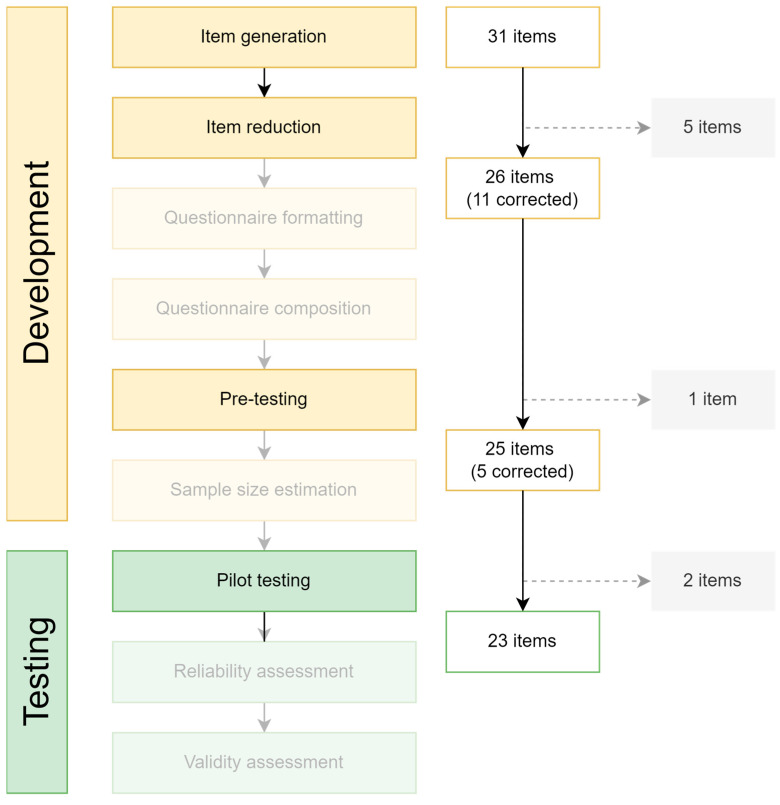
Generation, reduction, and correction of items throughout the questionnaire development.

An independent focus group assessed the list of generated items, choosing to remove five items and correct eleven items (Figure 3).

### 3.2. Pre-Testing

Individual interviews with 20 radiologists, similar to the sampling frame, did not identify major cognitive biases in the questions’ interpretation. However, we removed one question due to ambiguous perception and rephrased five items (Figure 3).

### 3.3. Pilot Testing

We distributed a web-based questionnaire form to the randomly chosen sample of Moscow radiologists. In total, 90 respondents filled out the questionnaire: 65 (72%) from MHD medical centers and 25 (28%) from MRC. All the questions were mandatory, so the obtained data had no missing values.

Among the respondents, there were 3 (3%) heads of radiology departments, 72 (80%) radiologists, and 15 (17%) radiology residents. Twenty-five (28%) respondents indicated that they participate in medical research activities. Among 63 (70%) respondents with 1+ years of professional experience, the median experience was 7 years (IQR 3 to 12 years). Three equal groups of respondents in the sample provided interpretations of a single imaging modality (usually computed tomography, CT), two modalities (the most common combination was radiography and CT), and three or more modalities, respectively. There were 57 (63%) and 10 (9%) adult and pediatric radiologists, respectively, with the rest interpreting studies of patients of all ages. Sixty-nine (77%) respondents indicated they have access to medical AI tools.

The majority of questions had substantial correlations within their domain. However, three questions showed weak correlation within their own domain and strong relationships with items from other domains. After the discussion with the expert group, two of these items were removed, and one was relocated to the appropriate domain. The final ATRAI-14 version has 23 questions composed of 14 main part items and 9 background part items.

**Figure 4:**
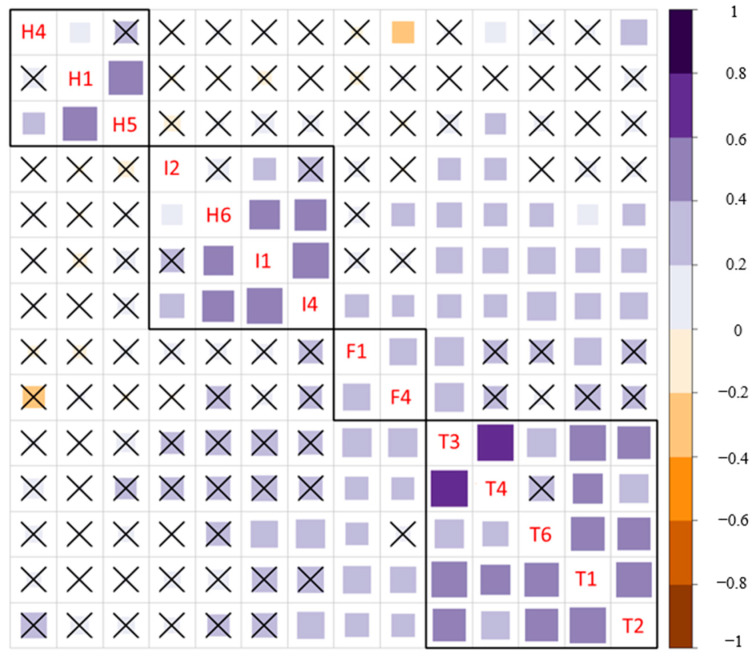
Questionnaire correlation matrix. ☓ marks beyond the diagonal denote statistical significance without multiple comparison correction. ☓ marks above the diagonal denote statistical significance with Holm–Bonferroni correction for multiple comparisons. Black outlined squares highlight automatically detected domains (from up to bottom): “Hopes and Fears” (items with “H” prefix), “Implementation Perspectives” (prefix “I”), “Familiarity” (prefix “F”), “Trust” (prefix “T”). Brown boxes represent negative correlation; blue boxes represent positive correlation.

According to the correlation matrix (Figure 4), there was no significant negative correlation between items from the domains contributing to scoring (“Trust”, “Implementation Perspective”, and “Hopes and Fears”). Correlation analysis confirmed that all the items measure the attitude in the same direction.

There was a weak significant correlation between items from the domains “Implementation Perspectives” and “Trust” (Figure 4). However, within these domains, the items had correlation strength varying from moderate to strong (Figure 4), which implies the correct distribution of items between the domains.

A weight adjustment was performed to compensate for the removal of items from the domains “Implementation Perspectives” and “Hopes and Fears” (Appendix A.2).

### 3.4. Validity

#### 3.4.1. Face Validity and Content Validity

According to the survey researchers and expert group assessment, all questions of the final version of the ATRAI-14 questionnaire were considered valid.

#### 3.4.2. Construct Validity

Confirmatory factor analysis (CFA) demonstrated that item loadings within a four-factor structure yield appropriate goodness-of-fit indices: RMSEA = 0.049, CFI = 0.95, TLI = 0.93, and SRMR = 0.067. For comparison, we performed CFA using a one-factor model, which demonstrated a worse fit with indices RMSEA = 0.11, CFI = 0.72, TLI = 0.66, and SRMR = 0.10. Item loadings of 13 out of 14 questions exceeded 0.55, demonstrating high adequacy of the factors and implying a good fit of the four-factor model (Table 2). Question H4 had the lowest loading score of 0.35 out of all items. However, this score still represents significant correspondence of the item to its domain. Thus, CFA results were consistent with the results of correlation analysis, supporting our assumption of the questionnaire’s four-domain structure.

#### 3.4.3. Criterion Validity

**Figure 5:**
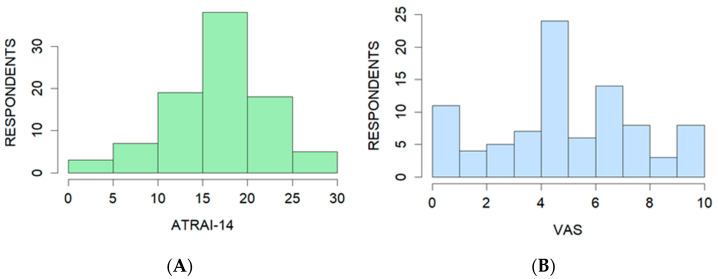
Distribution of the results of ATRAI-14 (**A**) and VAS (**B**) for 90 respondents.

The median ATRAI-14 score was 17.3 points (IQR 13.6 to 20.18 points), with a minimum of 2 points and a maximum of 27.5 points (Figure 5A). The median self-assessment according to the VAS was 5 points (IQR 4 to 7 points) (Figure 5B).

**Figure 6:**
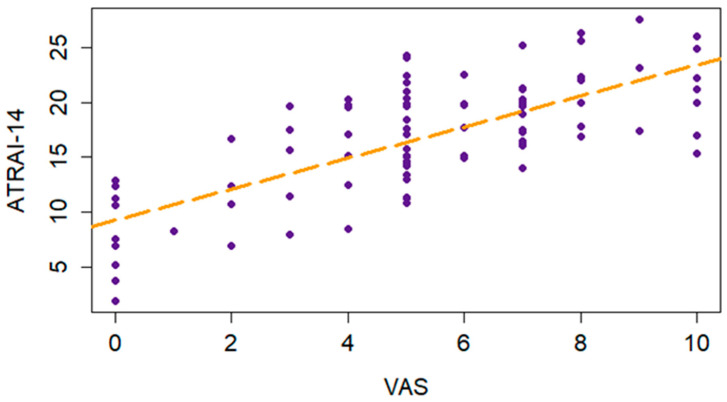
Scatter plot of the ATRAI-14 and VAS scores. The orange dotted line indicates the trend.

The correlation analysis demonstrated that the ATRAI-14 score had a strong correlation (Spearman’s rho 0.73, *p*-value < 0.001) with the self-reported attitude toward AI assessed by the VAS (Figure 6). These results support the measurement functioning of ATRAI-14.

### 3.5. Reliability

ATRAI-14 demonstrated good test–retest reliability (ICC = 0.89, CI [0.67; 0.96], *p*-value < 0.05) and acceptable internal consistency (Cronbach’s Alpha 0.78 95%CI [0.68, 0.83]).

## 4. Discussion

We developed and validated a questionnaire for the evaluation of radiologists’ attitude toward radiology AI (ATRAI-14), comprising a background part (9 questions related to a respondent’s demographics and professional characteristics) and 14 questions of the main part allocated into four domains: “Familiarity”, “Trust”, “Implementation Perspective”, and “Hopes and Fears”. Validation study results confirmed the high adequacy of the four-factor model, with item loadings of 13 out of 14 questions exceeding 0.55. ATRAI-14 has a high accuracy of attitude measurement (Spearman’s rho 0.73 with self-reported attitude toward AI assessed by the VAS); acceptable internal validity (Cronbach’s Alpha 0.78, 95%CI [0.68, 0.83]); and high test–retest reliability (ICC 0.89, 95%CI [0.67; 0.96], *p*-value < 0.05).

Staff attitude toward innovation can influence work behavior in scenarios where the innovation is used and ultimately determine the success of its implementation. There are several methodological issues specific to attitude research, one of which is a measurement error of self-reported measures of attitudes [36]. To evaluate the criterion validity of ATRAI-14, we used self-reported attitude toward AI assessed by the VAS (“Assess your attitude toward Radiology AI” with response options ranging from 0 to 10). We observed a strong positive correlation between ATRAI-14 and VAS scores (Spearman’s rho 0.73, *p*-value < 0.001), which implies that both instruments measure the attitude toward the same subject. Nevertheless, the relationships between the scores were not perfect. We believe that the difference is due to the four-domain structure of ATRAI-14 implicitly considering multiple aspects of a respondent’s attitude toward AI, therefore eliminating concerns with self-presentation and the lack of introspective access typical for self-reported measurements [22].

The “Familiarity” domain does not contribute to the total scoring but ranks the survey respondents by their experience with AI in three areas: clinical practice, development and testing, and participation in clinical trials. The final score of the questionnaire is a sum of the “Trust”, “Implementation Perspectives”, and “Hopes and Fears” weight-adjusted scores, with a maximum score of 36 (12 per domain).

The “Trust” domain measures subjective perceptions of the quality and reliability of current AI tools. We believe that media coverage of relevant, high-quality studies and practical workshops where radiologists can get hands-on experience can influence this domain.

The “Implementation Perspectives” domain reflects the respondent’s expectations regarding the mechanisms and outcomes of AI implementation. In our opinion, transparency of governmental policies toward AI is important for this domain.

The “Hopes and Fears” domain reflects a respondent’s perception of how AI will influence their career, including salary, workload, and occupational prestige. Fear of replacement is strongly associated with a respondent’s knowledge of AI [16], suggesting integrative education on AI as a valuable tool for influencing this domain.

Thus, the domain structure of ATRAI-14 is essential for determining the correspondence between the itemized attitude score and respondents’ background.

Follow-up surveys using ATRAI-14 can track changes in general attitudes toward AI and domain-specific dynamics at levels varying from the target population to individual radiologists. According to the survey results, administrative solutions aimed at successfully implementing AI could be specifically adapted. A target interaction seems possible for radiologists who are low on certain domains. Furthermore, group workshops can be conducted for radiologists with similar backgrounds and problematic domains.

Several attempts have been made to develop AI attitude assessment tools [15,16]. Codari et al. used a 21-item no-domain questionnaire designed by the European Society of Radiology (ESR). It had seven background items and fourteen main part items about respondents’ feelings and forecasts regarding the advent of AI applications in radiology [15]. Huisman et al. developed a 38-item questionnaire consisting of several domains to assess respondents’ views on AI implementation [16,17]. Both questionnaires did not have a scoring system and were not validated.

The key advantages of ATRAI-14 are (i) quantitative attitude assessment, (ii) good test–retest reliability and confirmed validity, and (iii) resolution ranging from a population level down to an individual radiologist. This study has several limitations. During the development and testing stages, we surveyed only MHD radiologists. We validated the questionnaire only for the Russian-speaking population. Finally, we introduced weight-adjusted scores for domains because of differences in the number of items. In future research, we plan to evaluate the influence of radiologist attitudes toward AI on clinical decisions made by the radiologist.

Radiology is one of the leading areas of applying technological advancements in medicine. Potential AI-driven changes in this field might require updating of the ATRAI-14 questionnaire in the future. 

## 5. Conclusions

Here, we present a questionnaire designed to measure the medical AI perception by radiologists across three domains: “Trust”, “Implementation Perspectives”, and “Hopes and Fears”. The questionnaire provides a precise estimation of the radiologists’ attitude toward AI in a resolution ranging from a population level down to individual healthcare professionals, distinguishing the tool from previous works in the field. The data we report confirm the construct validity of the questionnaire with high adequacy of the factors, acceptable internal consistency, and good test–retest reliability. The questionnaire is useful for providing detailed AI acceptance measurements of its end-users, which may be of particular value for making informed and directed management decisions when implementing AI-based software in radiology departments.

## Figures and Tables

**Table 1:** Methods used to assess reliability and validity.

Parameter	Method	Threshold Values
Test–retest reliability	Interclass Correlation Coefficient, ICC	<0.5—poor 0.5–0.75—moderate 0.75–0.9—good >0.9—excellent [23]
Internal consistency	Cronbach’s alpha	≤0.5—unacceptable >0.5—poor >0.6—questionable >0.7—acceptable >0.8—good >0.9—excellent [23]
Face validity	Experts evaluate whether the questionnaire measures what it intends to measure.	More than 75% of experts [28]
Content validity	Experts evaluate whether questionnaire content accurately assesses all fundamental aspects of the topic.	More than 75% of experts [28]
Construct validity	Confirmatory factor analysis	Comparative Fit Index (CFI) ≥ 0.9 Root Mean Square Error of Approximation (RSMEA) < 0.08 Standardized Root Mean Squared Residual (SRMR) < 0.08 Tucker–Lewis Index (TLI) ≥ 0.9 [29]
Criterion validity	Correlation with visual analogue scale	<0.10—negligible correlation 0.10–0.39—weak correlation 0.40–0.69—moderate correlation 0.70–0.89—strong correlation ≥0.90—very strong correlation [30]

**Table 2:** Factor loadings of the four-factor model.

Item	Standardized Factor Loadings (SE)				*p*-Value
	Factor 1 Familiarity	Factor 2 Trust	Factor 3 Implementation Perspective	Factor 4 Hopes and Fears	
F1	0.71 (0.16)	-	-	-	<0.001
F4	0.75 (0.18)	-	-	-	<0.001
T1	-	0.86 (0.11)	-	-	<0.001
T2	-	0.82 (0.12)	-	-	<0.001
T3	-	0.95 (0.12)	-	-	<0.001
T4	-	0.79 (0.13)	-	-	<0.001
T6	-	0.6 (0.1)	-	-	<0.001
I1	-	-	0.88 (0.13)	-	<0.001
I2	-	-	0.56 (0.19)	-	0.003
I4	-	-	0.86 (0.11)	-	<0.001
H6	-	-	0.75 (0.14)	-	<0.001
H1	-	-	-	0.67 (0.17)	<0.001
H4	-	-	-	0.35 (0.15)	0.022
H5	-	-	-	0.71 (0.16)	<0.001

## Data Availability

The original contributions presented in the study are included in the article, further inquiries can be directed to the corresponding author.

## Appendix A. ATRAI-14 Questionnaire

### Appendix A.1. Questionnaire

Radiologists’ Views on the Implementation of Artificial Intelligence in Diagnostic Imaging (ATRAI-14).

*Circles denote one answer questions, squares mark multiple answer questions.*

1. P1. State your job title (one answer)

Resident		○
Radiologist		○
Head of radiology department		○
Other (fill in)		○

2. P2. Working place (one answer)

University Hospital		○
Public Hospital		○
Teleradiology Center		○
Private Hospital		○
Other (fill in)		○

3. P3. State the type of medical facility you work at/study in (select all that applies)

Outpatient department	◻
In-patient department	◻
Day-case unit	◻

4. P4. What imaging modalities do you interpret on a daily basis? (select all that applies)

Radiography		◻
Mammography		◻
Ultrasound		◻
CT		◻
MRI		◻
Tomosynthesis		◻
Nuclear imaging		◻
Cone-beam CT		◻
Other (fill in)		◻

5. P5. What is the age of the patients you interpret studies for? (one answer)

<18 years old	○
≥18 years old	○
I work with patients of all ages	○

6. P6. State the anatomical regions/organ systems that you interpret most often (select all that applies)

I report all anatomical regions/organ systems	◻
Abdomen	◻
Thorax	◻
Breast	◻
Urogenital	◻
Musculoskeletal	◻
Neuroradiology	◻
Cardiovascular	◻
Pelvis	◻
Head and neck	◻

7. P7. State your total experience, in years, as a radiologist (excluding internship and residency)
8. P8. Do you partake in scientific research related to your medical activity? (one answer)

Yes	○
No	○

9. P9. Do you have the opportunity to use artificial intelligence (AI) tools at work to interpret imaging studies? (one answer)

Yes	○
No	○

Familiarity

10. F1. Do you use AI tools to interpret imaging studies? (one answer)

Answers:		label
Yes, regularly, for various tasks (example: for routine measurements or incidental findings detection)	○	4
Yes, regularly, a specific AI tool for a single task (example: to measure abdominal aorta diameter)	○	3
Yes, sometimes, depending on the task	○	2
Not yet, but I plan to	○	1
No, and I do not plan to	○	0

11. F4. How often do you participate in AI research projects? (one answer)

Answers:		label
Very often	○	4
Sometimes	○	3
Participated once or twice	○	2
Not yet, but I would want to	○	1
No, and I do not want to	○	0

Trust

12. T1. In the next 5 years, do you think you will be comfortable trusting autonomous AI to interpret imaging studies (an AI that acts without radiologist oversight)? (one answer)

Answers:		label
Yes	○	4
Mostly yes	○	3
Difficult to answer	○	2
Mostly no	○	1
No	○	0

13. T2. Do you trust the work of an AI tool outputting only «pathology present/study unremarkable»? (example: the presence or absence of liver lesions) (one answer)

Answers:		label
Yes	○	4
Mostly yes	○	3
Difficult to answer	○	2
Mostly no	○	1
No	○	0

14. T3. Do you trust the result of an AI tool outputting only a quantitative indicator (example: aortic diameter, pleural effusion volume, vertebral body height)? (one answer)

Answers:		label
Yes	○	4
Mostly yes	○	3
Difficult to answer	○	2
Mostly no	○	1
No	○	0

15. T4. Imagine—you read an imaging study and found no pathology, but an AI tool contradicts your opinion. Would this be reason enough for you to double check the study? (one answer)

Answers:		label
Yes	○	4
Mostly yes	○	3
Difficult to answer	○	2
Mostly no	○	1
No	○	0

16. T6. Which way of interacting with an AI tool would be preferable for you? (one answer)

Answers:		label
AI autonomously interprets a part of a study	○	4
AI performs routine measurements (diameter, volume, etc.) specified by an radiologist	○	1
AI filters out normal scans; the radiologist, without the help of an AI, analyzes only cases with suspected pathology	○	2
AI filters out normal scans; the radiologist, with the help of an AI, analyzes only cases with suspected pathology	○	3
AI is not included in image interpretation	○	0

Implementation Perspectives

17. I1. In your opinion, which of the listed functions of AI will be the most useful for radiologist? (select all that applies)

Answers:		label
Acceleration of the image reconstruction phase	◻	A
Image acquisition decision support system	◻	A
Image post-processing (quality improvement, noise reduction)	◻	A
Collection of quantitative data (size, volume, density/intensity)	◻	A
Incidental finding detection	◻	A
Cancer staging (as in TNM system)	◻	A
Assistance writing a structured report	◻	A
I don’t think AI can be useful	◻	B
I don’t think AI would be widely implemented	◻	B

18. I2. Imagine that usage of an AI is an additional healthcare service for a patient. Who, in your opinion, should pay for it? (one answer)

Answers:			label
Universal health care (compulsory medical insurance)		○	4
A patient’s insurance company		○	1
Hospital that hosts the AI		○	3
A patient		○	2
AI developer		○	1
I can not imagine such a situation		○	0
Other (fill in)	○	○	2

19. I4. In your opinion, what modalities will be most affected by AI in the next 5 years? (select all that applies)

Answers:		label
Radiography	◻	A
Mammography	◻	A
Ultrasound	◻	A
CT	◻	A
MRI	◻	A
Tomosynthesis	◻	A
Nuclear imaging	◻	A
I don’t think AI would have an impact	◻	B

20. H6. In your opinion, what will be the radiologist’s role in implementation of AI in medical imaging? (select all that applies)

Answers:		label
Image markup for AI training	◻	A
Formulating diagnostic tasks for developers	◻	A
Participation in the development of AI tools (programming, consulting for developers)	◻	A
Basic usability assessment (DICOM SR and SC) before implementation	◻	A
Pre-implementation diagnostic performance assessment	◻	A
Giving feedback on AI tools	◻	A
Radiologists will not be involved in any way in the development, testing or quality assessment of AI tools	◻	B
Difficult to say	◻	C

Hopes and Fears

21. H1. In your opinion, will the widespread use of AI by the radiologists affect the prestige of their profession in the next 5 years? (one answer)

Answers:		label
Will not affect/difficult to answer	○	2
Perhaps, the prestige will drop a little	○	1
Yes, the prestige will drop significantly	○	0
Perhaps, the prestige will grow a little	○	3
Yes, the prestige will grow significantly	○	4

22. H4. In your opinion, will the widespread use of AI affect the workload of the radiologists? (one answer)

Answers:		label
The workload will significantly decrease	○	4
The workload will somewhat decrease	○	3
It will not/difficult to say	○	2
The workload will somewhat increase	○	1
The workload will significantly increase	○	0

23. H5. In your opinion, will the widespread use of AI affect the salary of radiologists in your country in the next 5 years? (one answer)

Answers:		label
It will not/difficult to say	○	2
The salary will somewhat decrease	○	1
The salary will significantly decrease	○	0
The salary will somewhat increase	○	3
The salary will significantly increase	○	4

### Appendix A.2. Scoring

Questions from 1 to 9 are background questions and not scored.

For all questions from 10 to 23 (except for *I*1, *I*4, *H*6), the score corresponds to the number of the selected answer (listed under “label”).

For questions *I*1, *I*4, *H*6, multiple choice is implied. The sum of the selected answers’ individual scores is linearly converted to a Likert scale as follows:

*I*1: *B*—0, 1*A*—1, 2-3*A*—2, 4-5*A*—3, 6 or more *A*—4;
*I*4: *B* or 1*A*—4, 2-3*A*—3, 4-5*A*—2, 6*A*—1, 7*A*—0;
*H*6: *B*—0, *C*—1, 1-2*A*—2, 3-4*A*—3, 5-6*A*—4.

The “Familiarity” domain is scored as a sum of *F*1 and *F*4 questions.

The total score (S) of the questionnaire is calculated according to the equation:

*S* = *(T*1 + *T*2 + *T*3 + *T*4 + *T*6*)* * 0.6 *+ (I*1 *+ I*2 *+ I*4 *+ H*6*)* * 0.75 + *H*1 + *H*4 + *H*5
